# Assessment of Clinical Indicators Registered on Admission to the Hospital Related to Mortality Risk in Cancer Patients with COVID-19

**DOI:** 10.3390/jcm12030878

**Published:** 2023-01-22

**Authors:** Alina Szewczyk-Dąbrowska, Mirosław Banasik, Krystyna Dąbrowska, Krzysztof Kujawa, Wojciech Bombala, Agata Sebastian, Agnieszka Matera-Witkiewicz, Magdalena Krupińska, Urszula Grata-Borkowska, Janusz Sokołowski, Katarzyna Kiliś-Pstrusińska, Barbara Adamik, Adrian Doroszko, Krzysztof Kaliszewski, Michał Pomorski, Marcin Protasiewicz, Ewa A. Jankowska, Katarzyna Madziarska

**Affiliations:** 1 Department of Family Medicine, Wroclaw Medical University, Syrokomli Street 1, 51-141 Wroclaw, Poland; 2 Regional Specialist Hospital in Wroclaw, Research and Development Center, Kamieńskiego 73a, 51-124 Wroclaw, Poland; 3 Clinical Department of Nephrology and Transplantation Medicine, Wroclaw Medical University, Borowska Street 213, 50-556 Wroclaw, Poland; 4 Hirszfeld Institute of Immunology and Experimental Therapy, Weigla 12, 53-114 Wroclaw, Poland; 5 Statistical Analysis Centre, Wroclaw Medical University, K. Marcinkowski Street 2-6, 50-368 Wroclaw, Poland; 6 Clinical Department of Rheumatology and Internal Medicine, Wroclaw Medical University, Borowska Street 213, 50-556 Wroclaw, Poland; 7 Screening of Biological Activity Assays and Collection of Biological Material Laboratory, Wroclaw Medical University, Borowska Street 211a, 50-556 Wroclaw, Poland; 8 Clinical Department of Emergency Medicine, Wroclaw Medical University, Borowska Street 213, 50-556 Wroclaw, Poland; 9 Clinical Department of Paediatric Nephrology, Wroclaw Medical University, Borowska Street 213, 50-556 Wroclaw, Poland; 10 Clinical Department of Anaesthesiology and Intensive Therapy, Wroclaw Medical University, Borowska Street 213, 50-556 Wroclaw, Poland; 11 Clinical Department of Internal and Occupational Diseases, Hypertension and Clinical Oncology, Wroclaw Medical University, Borowska Street 213, 50-556 Wroclaw, Poland; 12 Clinical Department of General, Minimally Invasive and Endocrine Surgery, Wroclaw Medical University, Borowska Street 213, 50-556 Wroclaw, Poland; 13 Clinical Department of Gynecology and Obstetrics, Wroclaw Medical University, Borowska Street 213, 50-556 Wroclaw, Poland; 14 Institute of Heart Diseases, Wroclaw Medical University, Borowska Street 213, 50-556 Wroclaw, Poland; 15 Institute of Heart Diseases, University Hospital Wroclaw, Borowska Street 213, 50-556 Wroclaw, Poland

**Keywords:** COVID-19, infectious diseases, mortality risk, cancer, prognosis

## Abstract

Background: Oncology patients are a particularly vulnerable group to the severe course of COVID-19 due to, e.g., the suppression of the immune system. The study aimed to find links between parameters registered on admission to the hospital and the risk of later death in cancer patients with COVID-19. Methods: The study included patients with a reported history of malignant tumor (*n* = 151) and a control group with no history of cancer (*n* = 151) hospitalized due to COVID-19 between March 2020 and August 2021. The variables registered on admission were divided into categories for which we calculated the multivariate Cox proportional hazards models. Results: Multivariate Cox proportional hazards models were successfully obtained for the following categories: Patient data, Comorbidities, Signs recorded on admission, Medications used before hospitalization and Laboratory results recorded on admission. With the models developed for oncology patients, we identified the following variables that registered on patients’ admission were linked to significantly increased risk of death. They are: male sex, presence of metastases in neoplastic disease, impaired consciousness (somnolence or confusion), wheezes/rhonchi, the levels of white blood cells and neutrophils. Conclusion: Early identification of the indicators of a poorer prognosis may serve clinicians in better tailoring surveillance or treatment among cancer patients with COVID-19.

## 1. Introduction

The rapidly spreading SARS-CoV-2 virus has caused dramatic global difficulties, concern, and a death toll exceeding 6 million people so far [1]. In turn, cancer has been the second leading cause of death in the world for years [2]. Oncology patients are regarded as a population highly vulnerable to SARS-CoV-2 infection and the development of more severe COVID-19 symptoms, which is possibly due to the systemic immunosuppressive state caused by tumor growth and by the effects of anticancer treatment, as well as often observed poor health status in general [3,4]. Furthermore, there are several similarities between cancer and COVID-19 with respect to risk factors for severe disease, which have been elucidated by Zong et al. at the molecular level [5,6]. These include overproduction of cytokines (e.g., interleukin-6 [IL-6] and type I interferon [IFN-I]), involvement of androgen receptors and immune checkpoint signaling [5,6]. They have been shown to affect the incidence and severity of both diseases [5,6]. These reports emphasize the seriousness of COVID-19 infection in oncology patients.

Since the pandemic’s beginning, attempts have been made to identify the predictors of the course of infection [7,8,9]. The initially published studies were designed to support medical decisions. At the same time, the first prognostic models had a high risk of bias, mostly due to the unrepresentative selection of patients from the control group [8]. Among the data available to date, risk factors for death/severe disease in patients with COVID-19 have been shown to include age, male sex, smoking, and comorbidities including a coexisting cancer diagnosis [9,10].

The number of reports on prognostic factors focusing on oncology patients with COVID-19 is increasing and important factors identified so far, besides male sex and age, are metastatic cancer, renal impairment or heart disease [11,12,13]. The clinical features and epidemiology of COVID-19 differ widely among different countries and during different phases of the pandemic; therefore, further extending available data is the key for full understanding of the medical problem and for reliable predictions for all oncology patients [10]. Currently, when an increase in hospitalizations due to coronavirus infection is again being observed, a retrospective analysis of the variables recorded on hospital admission in a group of oncological patients with COVID-19 may help to identify risk factors of death. In practice, it will draw clinicians’ attention to specific signs and symptoms presented by a patient or indicated by laboratory diagnostics on admission, which may translate into more adequate treatment of the patient.

The aim of this study was to find links between the characteristics of cancer patients hospitalized for COVID-19, including diagnostic parameters registered on admission to the hospital, and the risk of later death in these patients. We aimed to improve the recognition of poor prognosis factors, the registration of which may be necessary for the care of this group of patients.

## 2. Materials and Methods

### 2.1. Study Design and Participants

The study was designed as a retrospective analysis of real-life cohorts. We analyzed medical records of patients hospitalized due to COVID-19 with a reported history of malignant tumor with or without metastases (*n* = 151). The control group consisted of 151 demographically matched people hospitalized due to COVID-19 with no history of cancer.

Data of all patients were collected between March 2020 and August 2021 and deposited in the password-protected database. The records were retrieved from the electronic medical records, which is the software operating in the University Hospital in Wroclaw and in the temporary hospital (operated from 9 March 2021 to 31 May 2021) run under the responsibility of the University Hospital.

Mortality data were obtained from electronic medical records and the Civil Registry Office (the governmental institution which registers births, marriages, and deaths in Poland) on 1 August 2021.

According to WHO recommendations, a positive nasopharyngeal swab obtained on admission determined by real-time reverse transcriptase-polymerase chain reaction (RT-PCR) or by an antigen testing was considered confirmed SARS-CoV-2 virus infection.

### 2.2. Ethical Statement

The study protocol was approved by the Institutional Review Board and Ethics Committee of Wroclaw Medical University, Wroclaw, Poland (No: KB-444/2021). The routine data were collected retrospectively and anonymized. The Bioethics Committee approved the publication of anonymized data.

### 2.3. Statistical Methods

In order to determine the factors (variables) affecting the risk of death, all patients (*n* = 302) were divided according to their outcome (dead or alive). The possible differences were established with Chi-square or Mann–Whitney U test, as appropriate, and the effect size calculated, using Vargha and Delaney’s A (K) for the Mann–Whitney test, and Odds Ratio for the Chi-square test [14]. A *p*-value < 0.05 was considered to determine the study-relevant variables exclusively, which were taken in further statistical analysis (Table S1).

Then, each variable registered on admission to the hospital was assigned to one of 6 categories: (i) patient data, (ii) comorbidities, (iii) signs (parameters measured/examined by a doctor) registered on admission, (iv) symptoms registered on admission, (v) medications used before hospitalization, (vi) laboratory results recorded on admission.

We analyzed the risk factors for death in both groups separately: Oncology Group: patients hospitalized for COVID-19 with cancer (*n* = 151); Non-Oncology Group: patients hospitalized for COVID-19 without cancer (control group) (*n* = 151).

For the categories patient data, comorbidities, symptoms recorded on admission, signs recorded on admission, medications used before hospitalization—the multivariate Cox proportional hazards models were calculated using the “all effects” method. For the category laboratory results recorded on admission, variables with low number of observations (<100) were excluded and we calculated the Cox model using “the best subset collection method” as a method of selecting variables. The proportionality assumption was met for all calculated Cox models. Finally, we generated graphs of the Breslow survival function.

Additionally, in order to find significant statistical differences between the variables registered on admission between the group of cancer patients and the group of patients without cancer, we performed the Chi-square or Mann–Whitney U tests.

Statistical analyses were performed using TIBCO Software Inc. (Palo Alto, CA, USA) (2017), Statistica (data analysis software system), version 13.

## 3. Results

### 3.1. Clinical Characteristics of Oncological Patients

Up to 1 August 2021, 151 patients with documented cancer were hospitalized due to COVID-19 at the University Hospital in Wroclaw.

The median age of these oncology patients was 68 (min–max: 17–95); 50.33% (76/151) were women. A hundred and ten patients (72.85%) had been diagnosed with a malignant tumor without metastases, and 41 of them (27.15%) had disseminated disease. Table 1 presents the clinical and demographic characteristics of the study groups. Table S2 contains the additional data on the clinical characteristics of oncology patients.

### 3.2. Factors Registered on Admission to the Hospital Affecting the Risk of Death

Multivariate Cox proportional hazards models were successfully obtained for the following categories: Patient data, Comorbidities, Signs recorded on admission, Medications used before hospitalization and Laboratory results recorded on admission (Table 2). With the models developed for oncology patients, we identified the following variables registered on patients’ admission that were linked to significantly increased risk of death. They are: male sex, presence of metastases in neoplastic disease, impaired consciousness (somnolence or confusion), wheezes/rhonchi, the levels of white blood cells (WBC) [10^3^/uL] and neutrophils [10^3^/uL] (Table 2).

**Figure 1:**
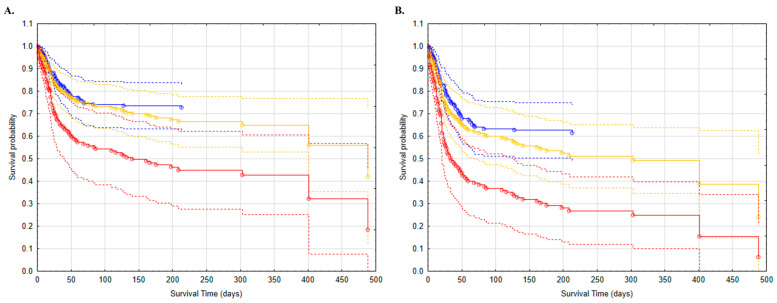
Survival analysis of patients with COVID-19: without cancer (blue); with cancer but without metastases (yellow); with cancer with metastases (red). (**A**) women; (**B**) men.

**Figure 2:**
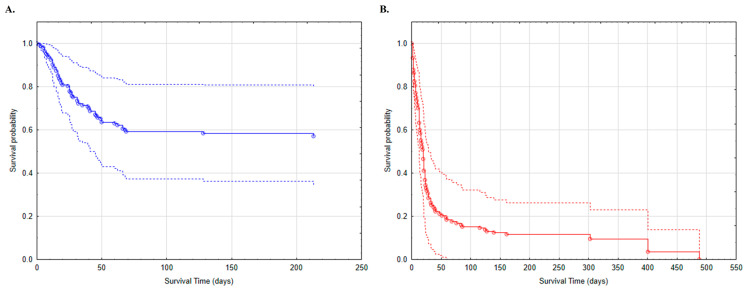
Survival analysis of patients with COVID-19 and impaired consciousness (somnolent/confusion) on admission to the hospital: (**A**) patients without cancer (blue); (**B**) patients with cancer (red).

**Figure 3:**
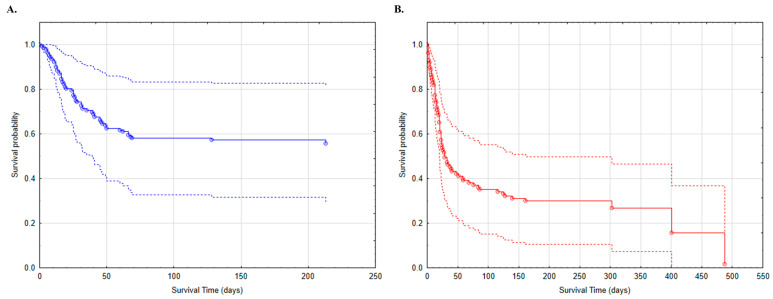
Survival analysis of patients with COVID-19 and wheezes/rhonchi on admission to the hospital: (**A**) patients without cancer (blue); (**B**) patients with cancer (red).

We also generated Breslow survival function graphs for the models (Figure 1, Figure 2 and Figure 3 and Figure S1A–G). Survival curves for non-cancer patients are shorter as no deaths have been recorded beyond the last marked data point. Figure 1, Figure 2 and Figure 3 show the variables that are the strongest predictors in our study: sex and the presence of metastases; wheezes/rhonchi; and impaired consciousness (somnolence or confusion) recorded on admission to the hospital.

## 4. Discussion

The World Health Organization has not announced the end of the pandemic so far, and there is more information about the growing number of COVID-19 infections in Europe (October 2022). The clinical picture of COVID-19 remains heterogeneous, and it is not entirely clear why some patients have a mild course of the disease and others are affected by a severe one [10]. Previous publications have shown that patients with malignant tumors are particularly vulnerable to severe disease, as evidenced by the activities of global societies of oncology updating their guidelines on an ongoing basis [15,16].

Cancer has already been described as an independent predictor of death in SARS-CoV-2 infected patients [10]. In a case–control study from an Italian group, the mortality of oncology patients with COVID-19 was significantly higher than those without neoplasm disease [10,17]. In our study, the Breslow plots for the variables assessed in our models indicate in most cases that oncology patients with COVID-19 have a lower chance of survival (Figure 1, Figure 2 and Figure 3 and Figure S1A–G). In addition, the presence of a metastatic solid tumor is a variable that significantly influences the risk of death (HR = 1.95), which means that the occurrence of metastases increases the risk of death by 95% compared to non-metastatic patients (Table 2). It should be noted that we did not analyze the direct cause of deaths, which in both groups could be the result of a diagnosed infection or comorbidities. However, previous reports show that patients with neoplasm, apart from being more susceptible to SARS-CoV-2 infections, are at an increased risk of more severe sequelae [13,18]. Furthermore, cancer itself is a heterogeneous disease, where, besides the treatment type, primary tumor subtype, age, sex and stage also play a role [19].

We observed that the male sex was a variable that increased the odds of death in the cancer group (HR = 0.61) (Table 2). This observation is consistent with scientific evidence showing male gender as a factor of death risk both in the general and in the oncologic populations with COVID-19 [9,10,13]. Our study demonstrates that being a woman reduces the risk of death by almost 40% in patients with neoplasm and coronavirus disease (Figure 1). The differences between males and females are observed not only in susceptibility to disease, but also in resilience to stress conditions and overall life expectancy [20]. Gender-related differences in the immune system, sex hormone environment and other unknown causes may be contributing factors to the high mortality rate of males under stressful conditions, including COVID-19 [20].

Other variables mentioned as factors of poor prognosis both among oncology patients and in general are age and comorbidities [11,21]. In our study, we successfully obtained the multivariate Cox proportional hazards model for comorbidities, but no disease was a variable that affected the risk of death in patients with both COVID-19 and cancer. In turn, age significantly influences the risk of death in our two investigated groups, therefore we do not present this variable as a cause of death in cancer patients; it represents a risk factor for patients with COVID-19 both with and without cancer. One of the more thorough systemic reviews conducted by Zhang et al. (2021) also indicates that sex (male) and age (elderly) appear to be risk factors associated with poorer prognostics in oncology patients [22]. In contrast to the oncology group, chronic kidney disease and intubation in the control group were found to be the indicators of poor prognosis.

Wheezes and rhonchi are signs of auscultation that may indicate inflammatory lesions in the lungs and are one of the criteria for the diagnosis of bronchitis or pneumonia [23,24]. The occurrence of the above symptoms in patients with COVID-19 infection may indicate severe respiratory disease [25]. We calculated that the presence of such symptoms on admission doubles the risk of death in our cancer patients, and the generated curve shows a lower survival chance in this COVID-19 patient group (Figure 3). The literature confirms that severe pneumonia is frequent in cancer patients with COVID-19 and leads to high mortality [26]. Although rales are considered the most common symptom at chest auscultation in COVID-19 patients [27] and were identified in 26 oncology patients (Table S2), they were not a variable affecting the risk of death (*p* = 0.709). Lung tumors are considered a risk factor for developing this condition [26]. A recent updated study of survival in patients with COVID-19 disease and lung cancer, which included more than 21,000 patients, found that lung cancer modifies COVID-19 prognosis in terms of disease progression and mortality [28]. However, among our patients, there were only 12 participants with primary lung cancer (Table S2) and we did not identify those who presented lung metastases, because the purpose of our study was to examine patients with any malignant tumors.

Impaired consciousness ranging from somnolence to confusion, delirium, stupor and coma has also been reported in patients with COVID-19 [29]. Moreover, there are reports that patients with more severe infections present symptoms such as impaired consciousness, and the study by Mao et al. (2020) suggests that clinicians, when seeing patients with neurologic manifestations, should suspect severe acute respiratory syndrome coronavirus infection to avoid delayed diagnosis [30]. According to our results, the occurrence of such symptoms significantly increases the risk of death in oncology patients (HR = 3.74) compared to the non-tumor group (Table 2, Figure 2). The remaining variables assessing consciousness are included in the Cox model, but do not represent risk factors of death in oncology patients (Table 2).

The analyses we performed showed an increased mortality risk in patients with elevated neutrophil count on admission. Oncological patients usually develop neutropenia during treatment with chemotherapy or as a result of comorbidities [31], but the release of neutrophil chemoattractant elements and the resulting neutrophil recruitment is a global host response to viral infection [32]. The analyses conducted so far have shown that COVID-19 patients present an increasing number of neutrophils during the severe phase [32]. Moreover, neutrophils as the marker of acute inflammation are associated with poor outcomes [33].

Both cancer and COVID-19 are related to increased risk of venous thromboembolic events, and D-dimer is considered a marker for early warning of disease severity and increased risk of death in patients with coronavirus infection [5,34]. In our study, D-dimer and the prothrombin index were variables affecting the risk of death (*p* = 0.0001 and *p* = 0.0420, respectively), but due to the number of observations they were not included in our model and, therefore, were not considered as factors of poor prognosis.

There are scientific reports on steroid therapy in patients with cancer and COVID-19 that indicate that continuing dexamethasone treatment after COVID-19 diagnosis may increase survival and improve prognosis in COVID-19, as opposed to discontinuing treatment after COVID-19 infection [35]. Among our oncology patients, only eight took oral steroids before hospitalization, seven of them continued treatment during hospitalization, and four died. Oral steroid use before hospitalization was not a variable affecting the risk of death in our patients (*p* > 0.05).

Importantly, this work was conducted as a registry of all cases hospitalized due to COVID-19 in the University Hospital in Wroclaw and in its branch (one of the most important temporary hospitals in Lower Silesia). Furthermore, multivariate Cox proportional hazards models developed herein showed results that were consistent with previous observations. The study, however, has a number of limitations that should be mentioned.

Firstly, we had no data available on cancer treatment, so there is a lack of analysis of treatment steps and disease activity; we also did not analyze the risk according to the type of cancer and the site of metastasis. Admittedly, Monnari et al. (2021) indicated that cancer patients, especially those with active cancer, have a higher risk of severe COVID-19 development; however, the research of Dai et al. (2020) shows that COVID-19 patients with both active treatment and just cancer history have a higher risk of developing severe events than non-cancer COVID-19 patients. They concluded that a malignant tumor had a lifetime effect on patients, and that cancer survivors need routine observation after primary treatment [12]. Furthermore, the analysis by Zhang et al. (2021) showed some interesting results. Primarily, there was no statistically significant difference in the increased risk of severe events in patients with lung cancer compared with other solid tumors [22]. Moreover, cancer patients receiving chemotherapy or other anticancer therapies did not show an increased risk of severe events with COVID-19 compared to patients not receiving active treatment [22]. Additionally, considering only patients with active cancer, the analysis showed that treatment (including chemotherapy, immunotherapy, targeted therapy, radiotherapy or surgery) was not associated with an increased risk of worse prognosis [22].

Secondly, the size of the study group and the number of data recorded on admission limited the possibility of analyzing the variables. This is due to the study’s retrospective design and the fact that most hospitalizations occurred during the second and third waves of the pandemic, where data input to the electronic system had a lower priority than patient survival. During these waves, a huge overload of the health care system occurred and patients were hospitalized in different wards not only with an internal medicine profile, including a temporary hospital, which became an important part of the strategy to fight the pandemic. A total of 270 doctors, 155 nurses, 100 doctors-in-training and many other medical workers who worked in various departments of the University Clinical Hospital on a daily basis were recruited to work there, and the involvement of physicians of various specialties provided comprehensive care to patients whose multiple diseases and clinical conditions required the cooperation of a multi-specialist team of medical professionals. In our facility, the approach to each patient was individualized, and only laboratory tests necessary to start the therapeutic process were ordered on admission.

In this research, as in other papers on similar topics, original analyses were performed, taking into account local specificity, a different set of predictors, and especially concomitant disease. For example, the study by Tiutan et al. (2022) showed that procalcitonin is a prognostic factor in cancer patients with COVID-19; however, this study did not take into account the survival time, and the observation time (death during or within 30 days of hospitalization) was also different [36]. Another interesting paper by Torres-Ruiz et al. (2021) also varied from our study, because each patient had the same set of lab tests performed on admission, blood samples were stored which improved the data availability, and the Cox model was used to predict both progression in COVID-19 and death, while we focused on death only [37].

In spite of these limitations, the results obtained in this study may provide valuable guidance on the course of hospitalization of cancer patients with COVID-19. We believe that the insights in this study will help to better understand the effects of SARS-CoV-2 infection in cancer patients.

## 5. Conclusions

In the face of the another observed increase in hospitalizations due to COVID-19 infection, early identification of diagnostic factors linked to poor prognosis may help clinicians in better tailoring surveillance or treatment among oncology patients with COVID-19. This study specifically revealed that these factors are: male sex, tumor metastasis, wheezes/rhonchi, and impaired consciousness (somnolence/confusion), as well as low white blood cell count and high neutrophils count registered on admission. Our results may help develop prognostic models or be used to compare the results of other studies, which will translate into improved treatment management, better prognosis and better care in this group of patients.

## Figures and Tables

**Table 1:** Clinical and demographic characteristics on oncological and non-oncological patients hospitalized due to COVID-19. The table presents only variables included in the study (variables affecting the risk of death with *p* < 0.05 are presented in Table S1).

	Patients with Cancer					Patients without Cancer					
Variable	*n*	Q1	Median	Q3	Average	*n*	Q1	Median	Q3	Average	*p* *
Patient data											
Alive (1 August 2021)	65/151	-	-	-	-	87/151	-	-	-	-	0.011
Female	76/151	-	-	-	-	76/151	-	-	-	-	1.000
Male	75/151	-	-	-	-	75/151	-	-	-	-	1.000
Age	151	61.00	69.00	78.00	67.78	151	61.00	69.00	78.00	67.78	1.000
Age ≥ 60 years	121/151	66.00	73.00	80.00	73.24	121/151	66.00	73.00	80.00	73.24	1.000
Age < 60 years	30/151	40.75	48.00	55.00	45.77	30/151	40.75	48.00	55.00	45.77	1.000
Comorbidities											
Previous Myocardial Infraction	20/151	-	-	-	-	24/151	-	-	-	-	0.514
Previous Coronary Revascularization	15/151	-	-	-	-	20/151	-	-	-	-	0.369
Asthma	2/151	-	-	-	-	9/151	-	-	-	-	0.032
Chronic Kidney Disease	18/151	-	-	-	-	27/151	-	-	-	-	0.146
Hemiplegia	5/151	-	-	-	-	11/151	-	-	-	-	0.123
Signs recorded on admission											
SBP [mmHg]	120	114.50	130.00	142.30	130.40	137	120.00	130.00	145.00	132.40	0.181
Impaired consciousness	17/150	-	-	-	-	21/151	-	-	-	-	0.501
Unconscious, not intubated	3/150	-	-	-	-	3/151	-	-	-	-	0.993
Unconscious, intubated	5/150	-	-	-	-	8/151	-	-	-	-	0.402
Wheezes/Rhonchi	22/151	-	-	-	-	15/151	-	-	-	-	0.219
Medication used before hospitalization											
LMWH	18/151	-	-	-	-	10/151	-	-	-	-	0.112
Laboratory results recorded on admission											
WBC [10^3^/uL]	149	5.90	7.41	10.50	10.46	151	5.51	7.46	11.67	11.01	0.795
Lymphocytes [10^3^/uL]	102	0.56	0.83	1.27	0.95	147	0.66	0.97	1.48	1.29	0.023
Neutrophils [10^3^/uL]	103	3.98	5.98	8.90	7.55	148	3.24	5.78	9.41	6.97	0.563
HGB [g/dL]	149	10.20	11.90	13.20	11.73	151	10.90	12.80	13.90	12.45	0.003
Potassium [mmol/L]	148	3.65	4.10	4.50	4.14	151	0.05	0.11	0.36	1.37	0.480
CRP [mg/L]	149	16.49	68.70	139.36	92.31	151	14.18	41.40	116.84	80.68	0.100
PCT [ng/mL]	105	0.07	0.18	0.72	1.35	145	0.05	0.11	0.36	1.37	0.037
INR	141	1.04	1.13	1.27	1.34	146	1.03	1.16	1.29	1.28	0.647
Urea [mg/dL]	143	29.00	41.00	66.00	57.34	151	29.00	44.00	72.00	58.25	0.671
Creatinine [mg/dL]	148	0.79	0.96	1.41	1.49	151	0.77	1.02	1.46	1.47	0.619
GFR [ml/min./1.73 m^2^]	148	46.50	70.50	90.00	70.66	150	42.00	67.00	94.00	68.79	0.597
Bilirubin [mg/dL]	115	0.50	0.60	1.00	1.18	140	0.40	0.60	0.90	0.78	0.409
GGTP [U/L]	108	24.50	40.50	120.00	124.30	141	25.00	42.00	87.00	73.56	0.301

*p* *—statistical difference between the group of cancer patients and the group of patients without cancer, calculated with the Chi-square or Mann–Whitney U tests. SBP: systolic blood pressure; LMWH: Low-molecular-weight heparin (standard prophylactic dose 4000 IU); WBC: White blood cells; HGB: Hemoglobin; CRP: C-reactive protein; PCT: Procalcitonin; INR: International Normalized Ratio; eGFR: Estimated Glomerular Filtration Rate; GGTP: Gamma-glutamyl Transferase.

**Table 2:** Multivariate Cox proportional hazards models. Significant factors marked with bold.

	Patients with Cancer				Patients without Cancer			
Variable	*p*	HR	95% CI HR Lower	95% CI HR Upper	*p*	HR	95% CI HR Lower	95% CI HR Upper
Model I—Patient data								
Age	**0.0009**	**1.030**	1.012	1.048	**0.0009**	**1.036**	1.015	1.058
Sex (F)	**0.0263**	**0.610**	0.394	0.943	0.0964	0.656	0.399	1.078
Tumor with metastases	**0.0029**	**1.959**	1.258	3.052	---	---	---	---
Model II—Comorbidities								
Previous Myocardial Infarction	0.0695	1.837	0.953	3.540	0.1616	1.677	0.813	3.461
Previous Coronary Revascularization	0.1904	1.682	0.772	3.664	0.9889	1.006	0.441	2.295
Asthma	0.7491	1.381	0.191	9.973	0.1153	0.202	0.028	1.478
Chronic Kidney Disease	0.4798	1.253	0.670	2.342	**0.0206**	**1.960**	1.109	3.464
Hemiplegia	0.3806	1.584	0.566	4.429	0.0763	1.983	0.930	4.226
Model III—Signs recorded on admission								
SBP	0.1014	0.992	0.982	1.002	0.3969	0.994	0.979	1.009
Impaired consciousness (somnolence or confusion)	**<0.0001**	**3.739**	1.997	7.002	0.7572	1.127	0.527	2.412
Unconscious, not intubated	0.1923	2.598	0.619	10.911	0.1128	3.749	0.732	19.195
Unconscious, intubated	0.3696	1.709	0.530	5.508	**0.0086**	**3.261**	1.350	7.876
Wheezes/Rhonchi	**0.0136**	**2.084**	1.163	3.732	0.7087	1.172	0.510	2.694
Model IV—Medications used before admission								
LMWH	0.0523	1.768	0.994	3.144	0.3115	1.544	0.666	3.583
Model V—Laboratory results recorded on admission								
WBC [10^3^/uL]	**0.0179**	**0.833**	0.716	0.969	0.1544	1.060	0.978	1.149
Lymphocytes [10^3^/uL]	0.7275	1.148	0.529	2.491	0.2290	0.747	0.465	1.201
Neutrophils [10^3^/uL]	**0.0058**	**1.263**	1.070	1.491	0.4346	0.960	0.867	1.063
HGB [g/dL]	0.7917	0.977	0.821	1.163	0.4266	0.950	0.837	1.078
Potassium [mmol/L]	0.2420	1.517	0.755	3.052	0.2254	1.345	0.833	2.172
CRP [mg/L]	0.0872	1.004	0.999	1.009	0.9194	1.000	0.996	1.004
PCT [ng/mL]	0.3510	1.049	0.948	1.162	0.0749	1.036	0.996	1.077
INR	0.9575	1.022	0.465	2.245	0.9108	0.978	0.663	1.442
Urea [mg/dL]	0.1288	1.008	0.998	1.019	0.5545	1.003	0.994	1.012
Creatinine [mg/dL]	0.1504	0.734	0.482	1.119	0.6836	1.070	0.773	1.481
GFR	0.7924	0.998	0.985	1.012	0.6550	0.997	0.985	1.010
Bilirubin [mg/dL]	0.1158	1.413	0.918	2.174	0.3690	1.283	0.745	2.208
GGTP [U/L]	0.4975	0.999	0.995	1.002	0.3276	1.002	0.998	1.005

SBP: systolic blood pressure; LMWH: Low-molecular-weight heparin (standard prophylactic dose 4000 IU); WBC: White blood cells; HGB: Haemoglobin; CRP: C-reactive protein; PCT: Procalcitonin; INR: International Normalized Ratio; eGFR: Estimated Glomerular Filtration Rate; GGTP: Gamma-glutamyl Transferase; CI: Confidence Interval. The *p* value was calculated in Cox models and is used to reject the null hypothesis that HR = 1.

## Data Availability

The datasets used and/or analyzed during the current study are available from the corresponding author upon reasonable request.

## Supplementary Materials

The following supporting information can be downloaded at: https://www.mdpi.com/article/10.3390/jcm12030878/s1, Table S1: The variables affecting the risk of death in patients hospitalized due to COVID-19; Table S2: Epidemiological and clinical characteristic of oncological patients hospitalized due to COVID-19; Figure S1: (A) Survival analysis of patients with COVID-19 and for choice values of laboratory results recorded on the admission to the hospital. (B) Survival analysis of patients with COVID-19 and previous myocardial infarction. (C) Survival analysis of patients with COVID-19 and previous coronary revascularization. (D) Survival analysis of patients with COVID-19 and asthma. (E) Survival analysis of patients with COVID-19 and Chronic Kidney Disease. (F) Survival analysis of patients with COVID-19 and hemiplegia. (G) Survival analysis of patients with COVID-19 and Low-molecular-weight heparin (LMWH) used before hospitalization.
