# Supplementary material for: Assessment of Clinical Indicators Registered on Admission to the Hospital Related to Mortality Risk in Cancer Patients with COVID-19
Source: J Clin Med. 2023 Jan 22;12(3):878. doi: 10.3390/jcm12030878 (PMC9917478; doi:10.3390/jcm12030878)
Supplement: Supplementary file 1 [file jcm-12-00878-s001.zip › jcm-2142288-supplementary.pdf]

Table S1. The variables affecting the risk of death in patients hospitalized due to COVID-19. Chi-square or Mann-Withney U test *p*-values are presented (only statistically significant variables). Effect size for numerical and categorical variables is expressed as Vargha and Delaney's A (VDA) and Odd Ratio (OR), respectively. Odd ratio is calculated for the level presented in parentheses at the variable name.

Variable	*p*	VDA/OR
Patient data		
Age [year]	<0.0001	0.65
Sex (Man)	0.0019	2.06
Tumor with or without metastases	<0.0001	na
Comorbidities		
Previous Myocardial Infarction (No)	0.0009	1.54
Previous Coronary Revascularization (No)	0.0434	2.11
Asthma (No)	0.0334	0.21
Chronic Kidney Disease (No)	0.0051	2.57
Hemiplegia (No)	0.0373	3.22
Signs recorded on admission		
SBP [mmHg]	0.0392	0.31
Consciousness on admission	0.0485	na
Wheezes/Rhonchi (No)	0.0201	2.33
Medication used before hospitalization		
LMWH (No)	0.0156	2.77
Laboratory results recorded on admission		
WBC [10*3/uL]	0.0015	0.60
Lymphocytes [10*3/uL]	0.0232	0.28
Neutrophiles [10*3/uL]	0.0001	0.44
HGB [g/dl]	0.0036	0.40
Potassium [mmol/l]	0.0081	0.58
CRP [mg/l]	0.007	0.58
PCT [ng/mL]	<0.0001	0.49
INR	0.0402	0.51
Urea [mg/dl]	<0.0001	0.65
Creatinine [mg/dl]	<0.0001	0.64
eGFR [ml/min.//1/73 m^2]	0.0002	0.36
Bilirubin [mg/dl]	0.0009	0.44
GGTP [U/L]	0.0157	0.40

VDA—Vargha and Delaney's A, OR—Odds Ratio; SBP: systolic blood pressure; LMWH: Low-molecular-weight heparin (standard prophylactic dose 4000 IU); WBC: White blood cells; HGB: Haemoglobin; CRP: C-reactive protein; PCT: Procalcitonin; INR: International Normalized Ratio; eGFR: Estimated Glomerular Filtration Rate; GGTP: Gamma-glutamyl Transferase, na—not applicable.

Table S2. Epidemiological and clinical characteristic of oncological patients hospitalized due to COVID-19.

Variable	*n*	%	Variable	*n*	%
Deaths up to 90 days from the admission	72/151	47.68%	**Symptoms and/or signs**		
Deaths up to 180 days from the admission	81/151	53.64%	Respiratory support on admission	59/151	39.07%
Number of patients transferred to ICU	10/151	6.62%	Dyspnoea	53/151	35.10%
**Type of cancer:**			Cough	31/151	20,53%
Colon	20/151	13.25%	Peripheral Oedema	28/151	18.54%
Recorded deaths	18/20	90.00%	Pleural effusion	26/151	17.22%
Breast	17/151	11.26%	Coarse Rales	26/151	17.22%
Recorded deaths	8/17	47.05%	Emesis	15/151	9.93%
Lung	12/151	7.95%	Abdominal Pain	12/151	7.95%
Recorded deaths	10/12	83.33%	Diarrhoea	11/151	7.28%
Prostate	12/151	7.95%	Chest Pain	10/151	6.62%

Recorded deaths	6/12	50.00%	Hipo-anosmia	2/151	1.32%
Stomach	12/151	7.95%	DBP > 90 [mmHg]	13/151	8.60%
Recorded deaths	10/12	83.33%	High Heart Rate	12/151	7.95%
Pancreas	10/151	6.62%	Oxygen therapy on admission	33/151	21.85%
Recorded deaths	5/10	50.00%	SaO2 < 95% without oxygen therapy	45/151	29.80%
Genitourinary	13/151	8.61%	SaO2 <95% with oxygen therapy	15/151	9.33%
Recorded deaths	7/13	53.85%	**Comorbidities**		
Ovarian	5/151	3.31%	Hypertension	83/151	54.97%
Recorded deaths	1/5	20.00%	Diabetes Mellitus	39/151	25.83%
Melanoma	3/151	1.99%	Atrial Fibrillation	21/151	13.91%
Recorded deaths	1/3	33.33%	Heart Failure	23/151	15.23%
Others	47/151	31.13%	Chronic Obstructive Pulmonary Disease	6/151	3.97%
Recorded deaths	22/47	46.81%	Previous Stroke	8/151	5.30%
**Previous therapies**			Smoking	24/151	15,89%
ACEI or ARB	47/151	31.12%	**Therapy during hospitalization**		
Betablockers	54/151	35.76%	Antibiotic therapy	110/151	72.85%
ASA	23/151	15.23%	Corticosteroids	71/151	47.02%
Statin	31/151	20.53%	LMWH	99/151	65.56%
Diuretic	36/151	23.84%	Remdesivir	18/151	11.92%
Oral steroid	8/151	5.30%	Convalescent plasma	13/151	8.61%
Immunosuppressive drug	8/151	5.30%			

ACEI: angiotensin-converting-enzyme inhibitors; ARB: angiotensin receptor blocker; ASA: acetylsalicylic acid; LMWH: Low-molecular-weight heparin (standard prophylactic dose 4000 IU); SaO2: oxygen saturation; PLT: Platelets; AST: aspartate aminotransferase; APTT: activated partial thromboplastin time, DBP: diastolic blood pressure, Comorbidities: chronic diseases that the patient suffers from in addition to cancer, ICU: Intensive Care Unit * Respiratory support on admission means any of the following: 1—passive oxygen therapy via oxygen sniffers, 2—straight face mask, 3—Venturi mask, 4—passive oxygen therapy via face mask with partially returnable or non-returnable reservoir bag, 5—high flow passive oxygen therapy (HFNC), 6—non-invasive ventilation (BiPAP/CPAP), 7—ventilator therapy (intubated or tracheostomy).

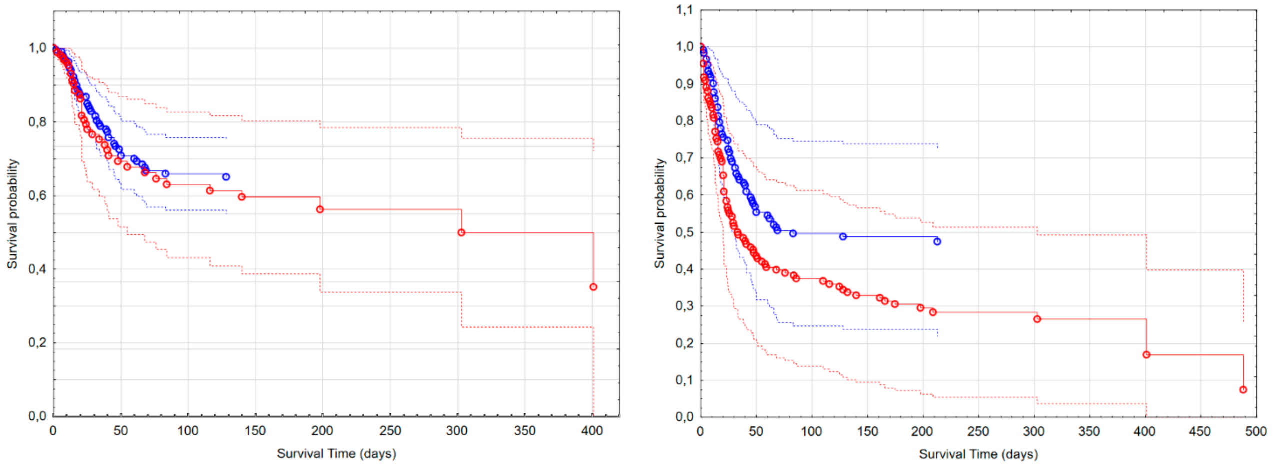

(**A**) (**B**)

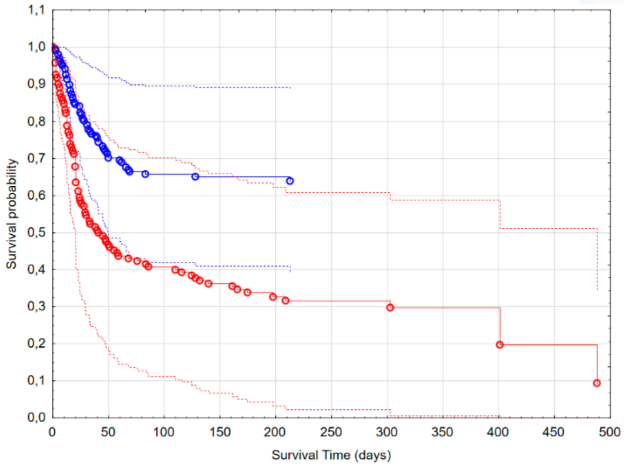

(C)

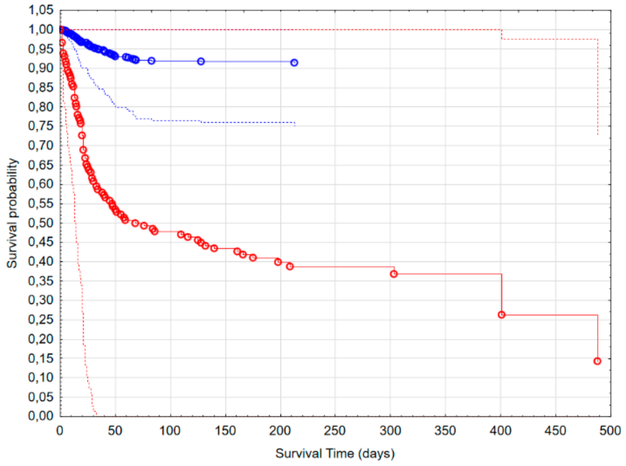

(D)

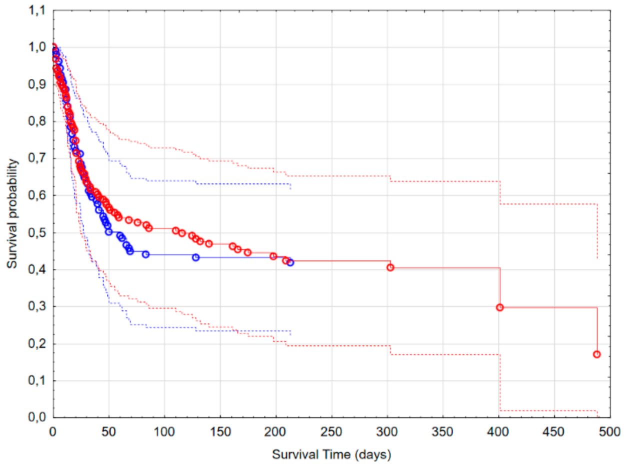

(E)

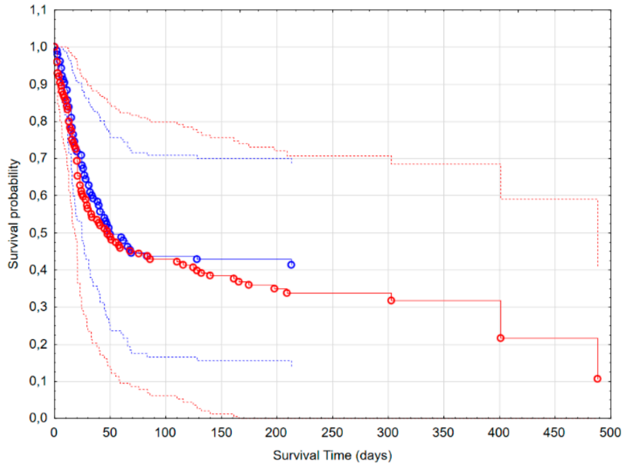

(F)

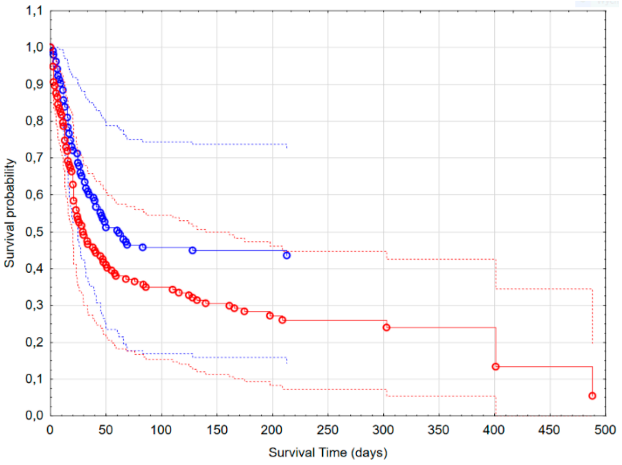

(G)

Figure S1. (**A**) Survival analysis of patients with COVID-19 and for choice values of laboratory results recorded on the admission to the hospital. Blue: patients without cancer; Red: patients with cancer. WBC [10*3/uL] = 10; Lympocytes [10*3/uL] = 1.5; Neutrophils [10*3/uL] = 7; HGB [g/dl] = 13; Potassium [mmol/L] = 4; CRP [mg/L]= 85; PCT [ng/mL] =1.5; INR = 1.3; Urea [mg/dl] = 58; Creatinine [mg/dl] = 1.5; eGFR [ml/min.//1/73 m2]= 69; Bilirubin [mg/dl] = 0.8; GGTP [U/L]= 78. (**B**) Survival analysis of patients with COVID-19 and previous myocardial infarction. Blue: patients without cancer; Red: patients with cancer. (**C**) Survival analysis of patients with COVID-19 and previous coronary revascularization. Blue: patients without cancer; Red: patients with cancer. (**D**) Survival analysis of patients with COVID-19 and asthma. Blue: patients without cancer; Red: patients with cancer. (E) Survival analysis of patients with COVID-19 and Chronic Kidney Disease. Blue: patients without cancer; Red: patients with cancer. (**F**) Survival analysis of patients with COVID-19 and hemiplegia. Blue: patients without cancer; Red: patients with cancer. (**G**) Survival analysis of patients

with COVID-19 and Low-molecular-weight heparin (LMWH) used before hospitalization. Blue: patients without cancer; Red: patients with cancer.
